# Effect of Phosphate on the Molecular Properties, Interactions, and Assembly of Engineered Spider Silk Proteins

**DOI:** 10.1021/acs.biomac.4c00115

**Published:** 2024-06-25

**Authors:** Yin Yin, Alessandra Griffo, Adrián Gutiérrez Cruz, Hendrik Hähl, Karin Jacobs, Markus B. Linder

**Affiliations:** † Department of Bioproducts and Biosystems, School of Chemical Engineering, Aalto University, Kemistintie 1, 02150 Espoo, Finland; ‡ Finnish Centre of Excellence in Life-Inspired Hybrid Materials (LIBER), Aalto University, Kemistintie 1, 02150 Espoo, Finland; § Biophysical Engineering Group, Max Planck Institute for Medical Research, 69120 Heidelberg, Germany; ∥ Department of Experimental Physics and Center for Biophysics, Saarland University, 66123 Saarbrücken, Germany; ⊥ Max Planck School “Matter to Life”, Jahnstraße 29, 69120 Heidelberg, Germany

## Abstract

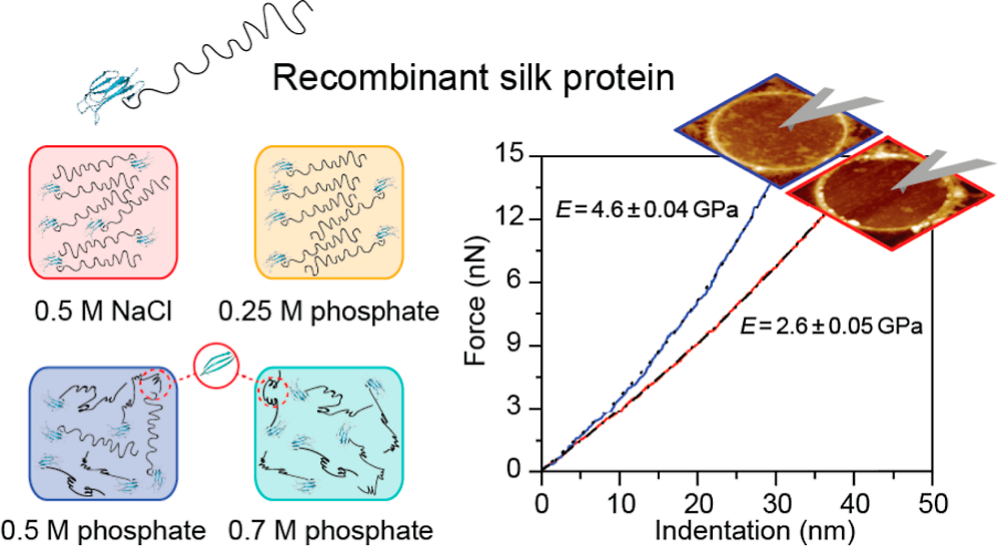

Phosphate plays a vital role in spider silk spinning and has been utilized in numerous artificial silk spinning attempts to replicate the remarkable mechanical properties of natural silk fiber. Its application in artificial processes has, however, yielded varying outcomes. It is thus necessary to investigate the origins and mechanisms behind these differences. By using recombinant silk protein SC-ADF3 derived from the garden spider *Araneus diadematus*, here, we describe its conformational changes under various conditions, elucidating the effect of phosphate on SC-ADF3 silk protein properties and interactions. Our results demonstrate that elevated phosphate levels induce the irreversible conformational conversion of SC-ADF3 from random coils to β-sheet structures, leading to decreased protein solubility over time. Furthermore, exposure of SC-ADF3 to phosphate stiffens already formed structures and reduces the ability to form new interactions. Our findings offer insights into the underlying mechanism through which phosphate-induced β-sheet structures in ADF3-related silk proteins impede fiber formation in the subsequent phases. From a broader perspective, our studies emphasize the significance of silk protein conformation for functional material formation, highlighting that the formation of β-sheet structures at the initial stages of protein assembly will affect the outcome of material forming processes.

## Introduction

**Figure 1:**
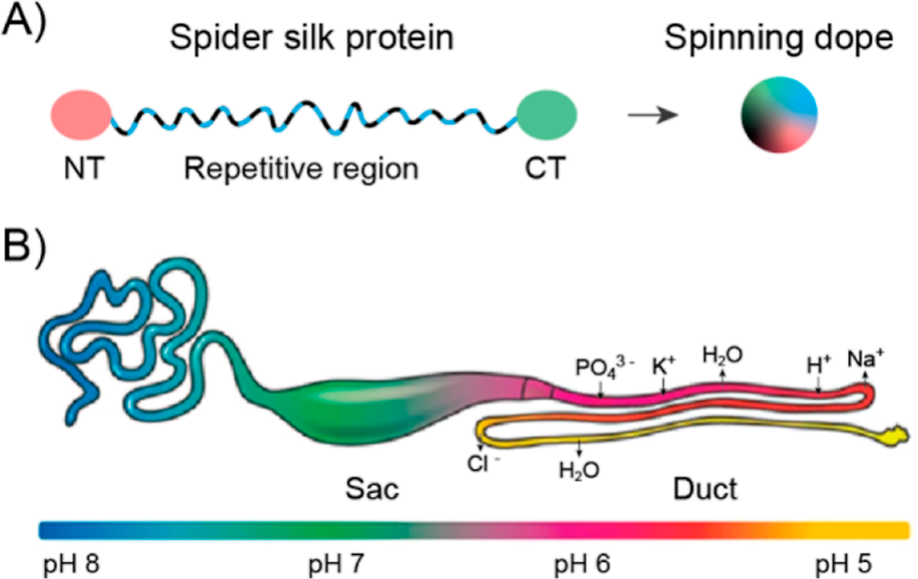
Illustration of a spider silk protein and a silk gland. (A) Canonical spider silk protein that contains a triblock structure and is stored as a highly concentrated spinning dope in the sac of the gland. (B) Spider silk gland. The spinning dope travels along the duct and turns into a β-sheet-rich solid thread through a very precise and controlled process. Panel B is adapted with permission from Rising and Johansson,^13^ Copyright 2015, Springer Nature.

Dragline silk fiber, spun from the major ampullate gland of the orb-weaving spider,^1−3^ possesses outstanding mechanical properties owing to highly hydrogen-bonded β-sheet nanocrystals that can form interlocking regions and facilitate stick–slip motions during stretching.^4,5^ The formation of the β-sheet structure in the dragline silk fiber is a complex and highly regulated process that occurs during silk spinning by these spiders. The dragline silk fiber is assembled from spidroins, which mainly contain two types of silk proteins that share a similar overall triblock structure characterized by a highly repetitive core region flanked by nonrepetitive N-terminal and C-terminal domains^6^ (Figure 1A). Before the spinning process, the spidroins are stored as a highly concentrated solution known as the “spinning dope”, where the repetitive sequence elements exist as a highly hydrated random coil and 3_1_-helix secondary structure.^7,8^ Through a very precise and controlled spinning process, the dope travels along the elongated S-shaped spinning duct and then turns into a β-sheet rich solid thread^1−3,9^ (Figure 1B). The secondary structure change from the random coil and 3_1_-helix in protein solution to β-sheets in the silk fiber can be induced through a combination of chemical and mechanical stimuli, such as decreasing the pH, exchanging ions, extracting water, extending flow, and shear forces.^10−12^

The kosmotropic ion phosphate, one of the ion compositions that increases as the dope travels down the duct,^12^ has a significant impact on the formation of β-sheet structures in silk proteins.^14−20^ This, in turn, affects silk protein behavior and the formation of silk fibers. Therefore, phosphate is involved in many studies that focus on mimicking spider silk to make artificial fibers. For example, in a study of chimeric major ampullate spidroin 2 protein (MaSp2) derived from *Trichonephila clavipes*, the addition of 0.5 M potassium phosphate triggered liquid–liquid phase separation (LLPS) or coacervation of the MaSp2.^21^ LLPS is a process that results in the coexistence of a dense phase and a dilute phase in a protein solution, with the protein-rich dense phase often referred as coacervates. Those phase-separated coacervates could form fibers upon manual stretching, which caused the appearance of β-sheets. The addition of 0.5 M potassium phosphate did not induce the protein conformational changes in either the phase-separated droplets or fibril network structures, as investigated by Raman spectroscopy. Another study on fibers made of highly concentrated eADF3, an engineered variant of the dragline silk proteins from the garden spider *Araneus diadematus*, revealed that the silk proteins existed in the presence of a low concentration of phosphate (30 to 50 mM) mainly in a random coil conformation.^22^ The phosphate ions led to the preordering of the silk protein over time and eventually resulted in LLPS within the eADF3 dope. The silk fibers pulled from this dope exhibited excellent strength and extensibility, with the resulting toughness comparable to that of native spider silk.^23^

In other studies, the existence of phosphate affected the final fiber formation. For the two engineered silk protein variants eADF3 and eADF4 from *A. diadematus*, 0.5 M potassium phosphate was shown to trigger the formation of microspheres through LLPS in these proteins.^15,24^ However, only spheres formed by eADF3 or mixtures of eADF3 and eADF4 induced by phosphate, together with elongational flow and pH drop, can assemble into fibers.^17^ This distinction arose from the conformational differences between eADF3 and eADF4 spheres, with eADF3 showing a dominant helical conformation and a conformational shift to the β-sheet structure during fiber formation, whereas the eADF4 sphere contained a dense packing of proteins that had a higher content of the β-sheet structure. Thus, it was suggested that eADF3 spheres possessed dangling ends that could mediate the interactions between adjacent assemblies, but eADF4 spheres lacked such ends that protruded to interact with other components.^17^

Similarly, our previous studies demonstrated that the presence of β-sheets induced by phosphate hinders subsequent fiber formation in air.^25^ Strong kosmotropes, such as potassium and phosphate ions, induced eADF3 proteins with a three-block architecture to form solid-like coacervates (SLCs), exhibiting properties distinct from liquid-like coacervates (LLCs) formed in pure water.^25^ The SLCs were typically smaller in size, denser in internal structure, much lower in protein diffusion rate, and unable to coalesce nor deform compared to LLCs. Notably, only LLCs could serve as intermediates for fiber formation, as no fiber formation was observed under any conditions for SLCs. The distinguishing features between LLCs and SLCs were attributed to the secondary structure conformation with LLCs having a high content of α-helices and SLCs experiencing a shift to β-sheet structures induced by phosphate. These findings suggest that metastable balanced molecular interactions, which are related to the repetitive regions in silk proteins, play a key role in subsequent structure formation.^19,25^

All of these studies underscore that the application of phosphate in artificial spinning demands meticulous control and caution despite its importance in spider silk spinning. This is because phosphate presents a substantial influence on silk proteins, especially on repetitive regions. Silk protein structures are highly dependent on the preparation methods, and specific states and interactions of these proteins are essential for fiber formation. Therefore, a comprehensive understanding of the effect of phosphate on the molecular properties and interactions of the repetitive regions of silk proteins will enhance the efficiency of artificial silk spinning development.

**Figure 2:**
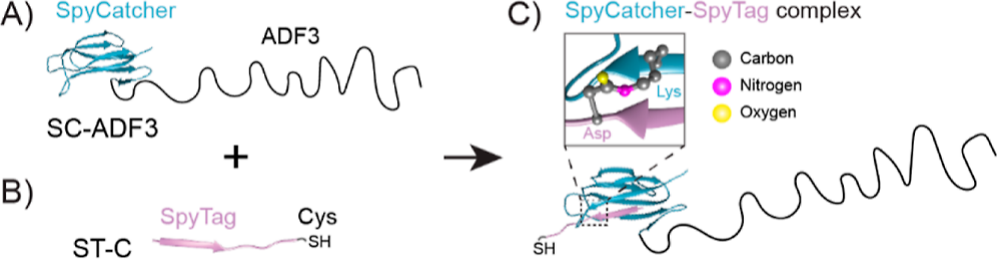
Schematic of the recombinant silk proteins used in this study. (A) SC-ADF3 contains a SpyCatcher domain (blue) and the repetitive sequence ADF3. (B) ST-C is a SpyTag peptide (purple) linked to a Cys residue. (C) SC-ADF3 and ST-C can spontaneously form an isopeptide covalent bond. Ribbon diagrams are based on PDB 4MLI.

To this end, we produced recombinant silk protein SpyCatcher-ADF3 (SC-ADF3) by fusing the repetitive sequence from *A. diadematus* (ADF3) with a SpyCatcher domain. The SpyCatcher domain in SC-ADF3 can react with a Cys-containing SpyTag (ST-C) peptide to form an isopeptide bond^26^ (Figure 2), and this reaction between SpyCatcher and SpyTag occurs under versatile conditions.^26−28^ The thiol group in Cys exhibits high affinity with gold,^29^ facilitating the immobilization of SC-ADF3 proteins on gold surfaces, enabling us to examine the conformational changes of the proteins under different experimental conditions.

By using circular dichroism (CD), quartz crystal microbalance with dissipation monitoring (QCM-D), and atomic force microscopy (AFM) indentation and AFM in force spectroscopy (FS) mode, we investigated the effects of different phosphate concentrations on the recombinant SC-ADF3 silk proteins. We found that increased phosphate levels caused an irreversible conformational change of the SC-ADF3 silk protein from random coils to β-sheets, resulting in a stiffer layer and a reduced ability to form new interactions. Therefore, those changes resulted in the lack of cohesion between protein molecules during deformation and it may prevent silk proteins from forming material structures. Our results elucidate the relationship between protein conformation and their final functional states.

## Materials and Methods

### Plasmid Construction

Genes encoding for SpyCatcher (an E48K variant of SpyCatcher^26^) and ADF3^30^ in SC-ADF3 construct were amplified by PCR using primers containing designed restriction sites. The amplified fragments were then cloned into the bacterial expression vector pET28 (+) in frame with the C-terminal 6xHis-tag through standard restriction digestion and ligation protocols. TOP10 was used as the cloning strain. The sequence of the plasmid was verified through Sanger sequencing from Eurofins. Sequence details are available in the Supporting Information.

### Protein Expression and Purification

BL21(DE3) was used as the expression strain, and MagicMedia *E. coli* expression medium (Thermo Fisher Scientific) was used for protein expression according to the protocol provided by the manufacturer. Protein expression was carried out at 30 °C and 220 rpm for 24 h. Cells were harvested by centrifuging at 5000 rpm for 20 min, resuspended in 5 mL per gram lysis buffer (50 mM tris-HCl pH 7.4, 0.1 M NaCl, 1 mg/mL lysozyme, 10 μg/mL DNaseI, 3 mM MgCl_2_, 1 tablet of protease inhibitor cocktail in 100 mL buffer), and incubated for 1 h. Cell disruption was achieved using a high-pressure homogenizer (AVESTIN-EmulsiFlex-C3). Cell debris was removed by centrifuging at 12,000 rpm for 20 min, and the protein-containing supernatant was loaded onto a HisTrap FF crude column (GE Healthcare Life Sciences) connected to an ÄKTA-Pure fast protein liquid chromatography system. A buffer containing 500 mM NaCl and 20 mM imidazole (pH 7.4) was used to wash the system and unbound proteins, while a buffer with 500 mM NaCl and 500 mM imidazole (pH 7.4) was used to elute the target proteins. Protein samples were desalted and concentrated using Econo-Pac 10DG desalting prepacked gravity flow columns (Bio-Rad) and Vivaspin 20, 30 kDa MWCO centrifugal concentrators (Sartorius), respectively. The final protein concentration was determined by a Varian Cary 50 UV–vis spectrophotometer. Protein purity was checked using sodium dodecyl sulfate–polyacrylamide gel electrophoresis (SDS-PAGE) (Figure S1). Aliquots of the protein samples were flash-frozen in liquid nitrogen and stored at −80 °C.

### Circular Dichroism

CD measurements were carried out on a JASCO J-1500 spectrometer to study the secondary structure of SC-ADF3 under various conditions. Each sample underwent 8 accumulated scans with baseline correction over a wavelength range from 260 to 190 nm using a 1 nm bandwidth at 22 °C for most of the measurements. For temperature dependence analysis, 3 spectra were recorded at 20 °C, followed by gradual temperature increments from 20 to 90 °C at 5 °C intervals, and finally remeasured at 20 °C after cooling down. High-tension spectra were simultaneously collected with CD spectra to monitor the data quality. Protein concentrations ranged from 0.1 to 0.28 mg/mL, as needed. A 1 mm path-length quartz cuvette (Hellma) was used, and all spectra were smoothed using the Savitzky–Golay filter with a point-window of 5 in Spectra Analysis software (JASCO). The CD spectra were represented in molar ellipticity [θ] (deg·cm^2^·dmol^–1^), according to eq 1
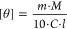
1where *m* is the CD signal (millidegrees), *M* is the average molecular weight (g/mol), *C* is the protein concentration (g/L), and *l* is the path length (cm).

### Light Microscopy

Proteins in various solutions were visually inspected for the presence of protein aggregates using an Axiovert inverted light microscope equipped with a 10 to 40×/1.6 objective (ZEISS).

### Quartz Crystal Microbalance with Dissipation Monitoring

QCM-D was utilized to investigate the viscoelastic properties of the adsorbed protein layer under various conditions. Prior to the adsorption process, a reaction between SC-ADF3 and ST-C was conducted, where the SpyCatcher domain from SC-ADF3 formed an isopeptide bond with SpyTag in the ST-C peptide through SpyCatcher–SpyTag ligation. The mixture containing 1 mg/mL SC-ADF3 and 0.028 mg/mL ST-C (molar ratio of SC/ST = 1) was incubated at 22 °C overnight for later use.

Q-Sense E4 and AT-cut QSX 301 gold-coated sensors (Biolin Scientific) were used for the QCM-D measurements at 22 °C using a flow rate of 50 μL/min. The protein solution containing a mixture of SC-ADF3 and ST-C was diluted to 0.5 mg/mL in 0.5 M NaCl before loading. A blank baseline was established by flowing 0.5 M NaCl through all chambers for 10 min. The protein solutions in 0.5 M NaCl were flowed over the gold sensors in different chambers for approximately 2 h, followed by rinsing with 0.5 M NaCl to wash away unbound proteins for 20 min. Subsequently, 0.25, 0.5, and 0.7 M sodium phosphate (pH 7.4) were flowed into different chambers, respectively. After 1 h and 40 min, all chambers were flushed with 0.5 M NaCl again. During the measurements, the changes in the resonance frequency (Δ*f*) and energy dissipation (Δ*D*) were monitored. The measurements were performed in triplicate. The relative changes in the ratio of Δ*D*/|Δ*f*| after exposure to different concentrations of sodium phosphate were calculated by , where *R* = Δ*D*/|Δ*f*|.

### AFM Indentation Experiments

For the indentation experiments, silk layers were transferred onto holey substrates (QUANTIFOIL Holey Carbon supports, Quantifoil Micro Tools GmbH) using a lift-up transfer method. In detail, the carbon support consisted of carbon films of 10–12 nm thickness with 1.2 μm diameter holes, arranged in a regular pattern with distances of 1.3 μm. The underlying TEM grid structure consisted of a copper mesh. The silk layers were prepared through self-assembly of SC-ADF3 molecules at the air–water interface as similarly reported in earlier works.^31,32^ A glass beaker (ø 4 cm) containing 0.5 μM SC-ADF3 in the buffer of interest was left in the fridge overnight to guarantee full coverage of the self-assembled monolayer at the air–water interface. The lift-up transfer method, which consisted of horizontally lifting upward through the floating protein monolayer of the QUANTIFOIL grid, was used to deposit the as-prepared layers on top of the surfaces of interest. After the transfer and an adsorption time of 10 min, the samples were rinsed gently to remove excess unabsorbed protein. The protein layers can also be transferred onto a smooth, hydrophobic surface in order to characterize their thickness and topography. For that, we prepared a self-assembled monolayer of octadecyltrichlorosilane on Si wafers (OTS-Si)^33^ to obtain information on layer thickness, which is needed for the determination of the elastic modulus of the indentation experiments via the chosen elastic model.

Indentation experiments were carried out using an AFM FastScan Bio Instrument (Bruker) with SCANASYST-AIR Bruker tips (0.4 N/m spring constant, tip radius of 2 nm). The spring constant was determined in air by using the thermal tune method. Several indentation cycles were measured in the middle of the free-standing silk-covered hole with trigger thresholds ranging from 2 to 20 nN, until the layer ruptured. Indentation curves were recorded and fitted using the elasticity model for films clamped onto a circular hole
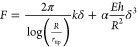

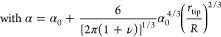
2where *h* is the layer thickness assumed according to the AFM images of protein layers on OTS-Si substrates (Figure S2), *R* is the hole radius, *E* is the elastic modulus, *k* is the prestrain of the membrane, and α_0_ ≈ 0.867 + 0.2773ν + 0.8052ν^2^.^34^

### AFM Imaging

Olympus (OMCL-TR800PSA) tips were used for AFM imaging, Bruker SCANASYST-FLUID tips were used for analysis in liquid, and Bruker SCANASYST-AIR tips were used for the indentation analysis. Peak force mode was used for imaging in air and liquid at different scan sizes (10.0, 5.0, 3.3, 1.7, and 0.5 μm) and rates (0.5–1.0 Hz). The NanoScope Analysis program was used to flatten the images and remove tilts.

### Single-Molecule Force Spectroscopy Experiments

For AFM tip functionalization, a two-step process was employed. First, an AFM gold cantilever (CSG 11/Au 0.03 N/m spring constant, Pra·Ma) was functionalized with ST-C. Then, a solution containing 0.5 μM SC-ADF3 was drop-casted on the ST-C-coated tip and allowed to react for 15 min. The functionalized tip was rinsed twice with the buffer of interest to remove unbound proteins. The functionalized tips were prepared and used on the same day as the SMFS experiments. For surface functionalization on gold QCM-D sensors, the same procedure as tip functionalization was followed, except that the concentration of SC-ADF3 was increased to 1 μM.

The SMFS experiments were carried out with a Bruker MultiMode 8 instrument, with the sample immersed in 0.5 M sodium phosphate (pH 7.4) or 0.5 M NaCl. At least 500–600 single force/distance curves were recorded and analyzed for curves featuring specific adhesion. The force measurements were conducted using a ramp size of 500 nm, a scan rate of 0.5 Hz, a forward and reverse velocity of 200 and 500 nm/s, and a relative force trigger of 260 pN. Two different series were performed, at forward and reverse velocity of either 200 or 500 nm/s. The data was processed using homemade MATLAB scripts for baseline and contact point correction and extraction of the rupture force of the first adhesion signal. The spring constant was determined in liquid by using the thermal tune technique.

## Results

### Higher Phosphate Concentration Led to More β-Sheet Structure Formation

**Figure 3:**
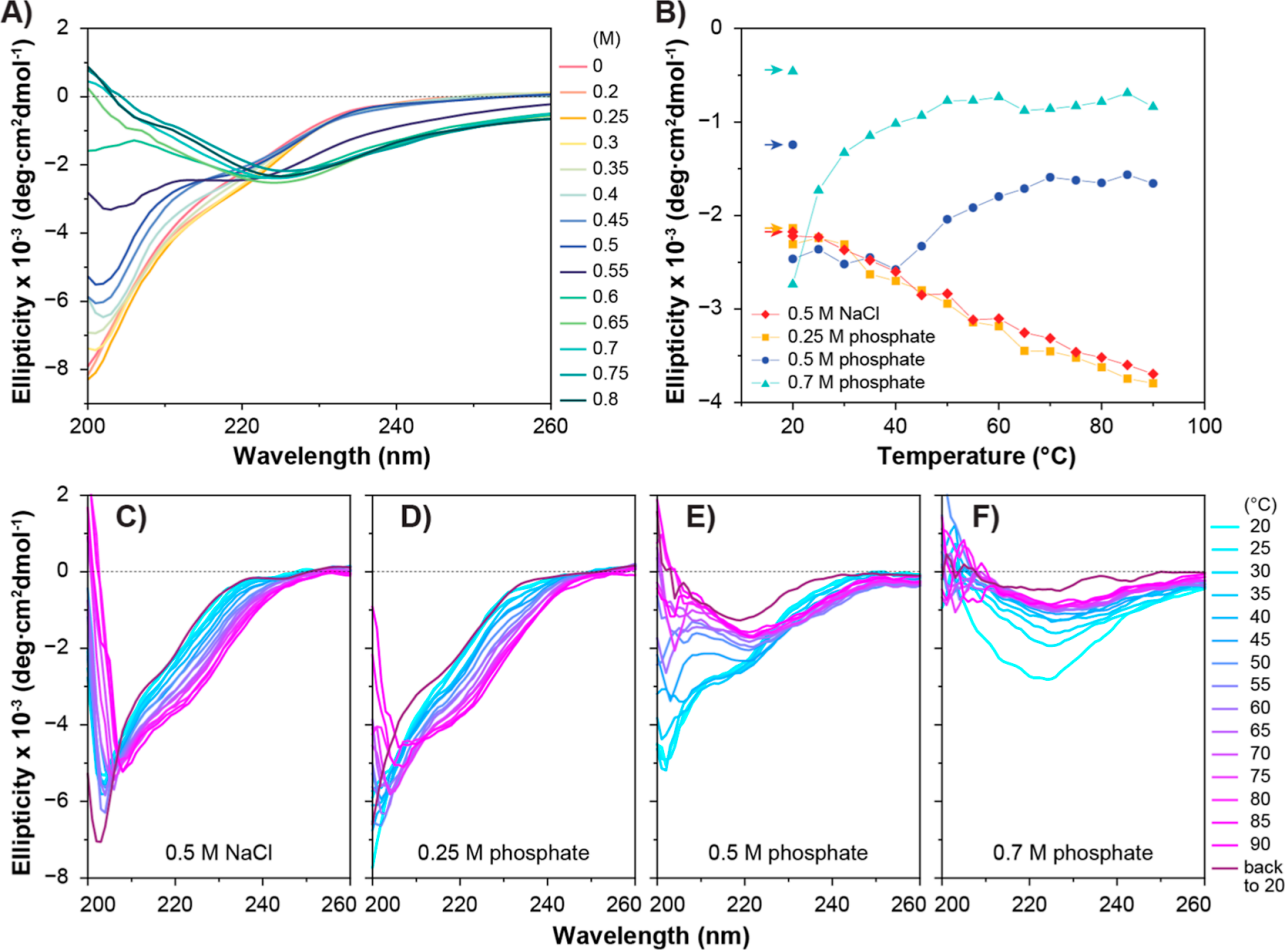
Effects of varying phosphate concentrations on the secondary structure of SC-ADF3. (A) CD spectra of 0.1 mg/mL SC-ADF3 in different concentrations of sodium phosphate (pH 7.4) at 22 °C. (B–F) Temperature dependence of SC-ADF3 in 0.5 M sodium chloride and 0.25, 0.5, and 0.7 M sodium phosphate. Molar ellipticities of SC-ADF3 were measured at 220 nm while heating from 20 to 90 °C and then cooling back to 20 °C (indicated by arrows) in (B) and CD spectra of SC-ADF3 were recorded during the temperature ramp from 20 to 90 °C and then back to 20 °C in (C–F).

The effects of varying sodium phosphate concentrations on the secondary structure of SC-ADF3 were first investigated using CD spectroscopy (Figure 3A). In the absence of phosphate (0 M), the CD spectrum exhibited characteristics of a random coil-dominated secondary structure. As the sodium phosphate concentration increased from 0.2 to 0.8 M, the CD spectra of SC-ADF3 showed a loss of random coil features, and a peak emerged at a wavelength range of 225–230 nm, indicating the presence of a distinct β-sheet structure. The most significant change occurred between 0.45 and 0.6 M phosphate concentrations, whereas concentrations below 0.35 M had minimal impact on the secondary structure of SC-ADF3. At 0.5 M sodium phosphate, the spectra displayed broader minima centered at 219 nm, indicative of contributions from both random coil and β-sheet structures. Additionally, above 0.65 M sodium phosphate, the random coil propensity decreased, signifying a reduction in random coil content. The transition to β-sheet structures by increase in phosphate concentrations was verified using Fourier-transform infrared spectroscopy (FTIR) by showing a characteristic shift in the amide I band from 1642 to 1620 cm^–1^ (Figure S3). Our interpretation of secondary structure changes in ADF3 based on CD spectra and FTIR is supported by previous analysis of similar silk proteins.^19,35,36^ The conformational changes induced by phosphate were attributed primarily to the silk part (ADF3) rather than the SpyCatcher (SC), as the SC spectra remained similar regardless of the presence of 0.8 M sodium phosphate (Figure S4). Furthermore, in contrast to sodium phosphate, the addition of 0.8 M sodium chloride had a minimal impact on the CD spectrum (Figure S4). To study the possible influence of SC on secondary structure changes, we verified that a similar response in phosphate-induced secondary structure changes was also observed in another ADF3-containing protein, CBM-ADF3-CBM, which is flanked by cellulose-binding modules (CBMs) at the N- and C-termini (Figure S5). Therefore, we conclude that the CD measurements revealed an increase in the β-sheet secondary structure of ADF3 in SC-ADF3 with increasing phosphate concentrations.

### Increased Temperatures Induced Structural Changes in Higher Phosphate Concentrations

Subsequently, we investigated changes in the secondary structure of SC-ADF3 over a range of temperatures in the presence of phosphate (Figure 3B–F). SC-ADF3 in 0.5 M sodium chloride was also studied as a control group (Figure 3C). In the presence of 0.25 M sodium phosphate at 20 °C, SC-ADF3 exhibited a predominantly random coil structure, similar to its conformation in 0.5 M sodium chloride (Figure 3D). Cooperative temperature transitions were not observed in either 0.5 M sodium chloride or 0.25 M sodium phosphate, and the temperature-induced conformational change was fully reversible upon heating and cooling. In the presence of 0.5 M sodium phosphate (Figure 3E), we observed a transition of SC-ADF3 to a β-sheet structure with an increasing temperature. SC-ADF3 remained stable up to 40 °C, displaying a combination of the random coil and β-sheet structure. Above 40 °C, SC-ADF3 underwent an irreversible structural conversion, ultimately adopting a predominantly β-sheet state. In 0.7 M phosphate (Figure 3F), SC-ADF3 exhibited a primarily β-sheet structure at 20 °C and displayed a conformational change with transition temperatures at 25 °C. This thermal conformational change process was irreversible upon cooling. The same irreversibility was observed also when heating samples to a lower temperature of 50 °C. Heating 0.7 M sodium phosphate led to reduced absolute molar ellipticity and slight opaqueness, suggesting protein aggregation and precipitation. These results showed that increased temperatures induced irreversible structural changes of SC-ADF3 with a β-sheet structure induced by phosphate. At 22 °C, the solutions remained clear, and it was selected as the experimental temperature for subsequent studies.

### β-Sheet Structure Formation is Irreversible over Time

**Figure 4:**
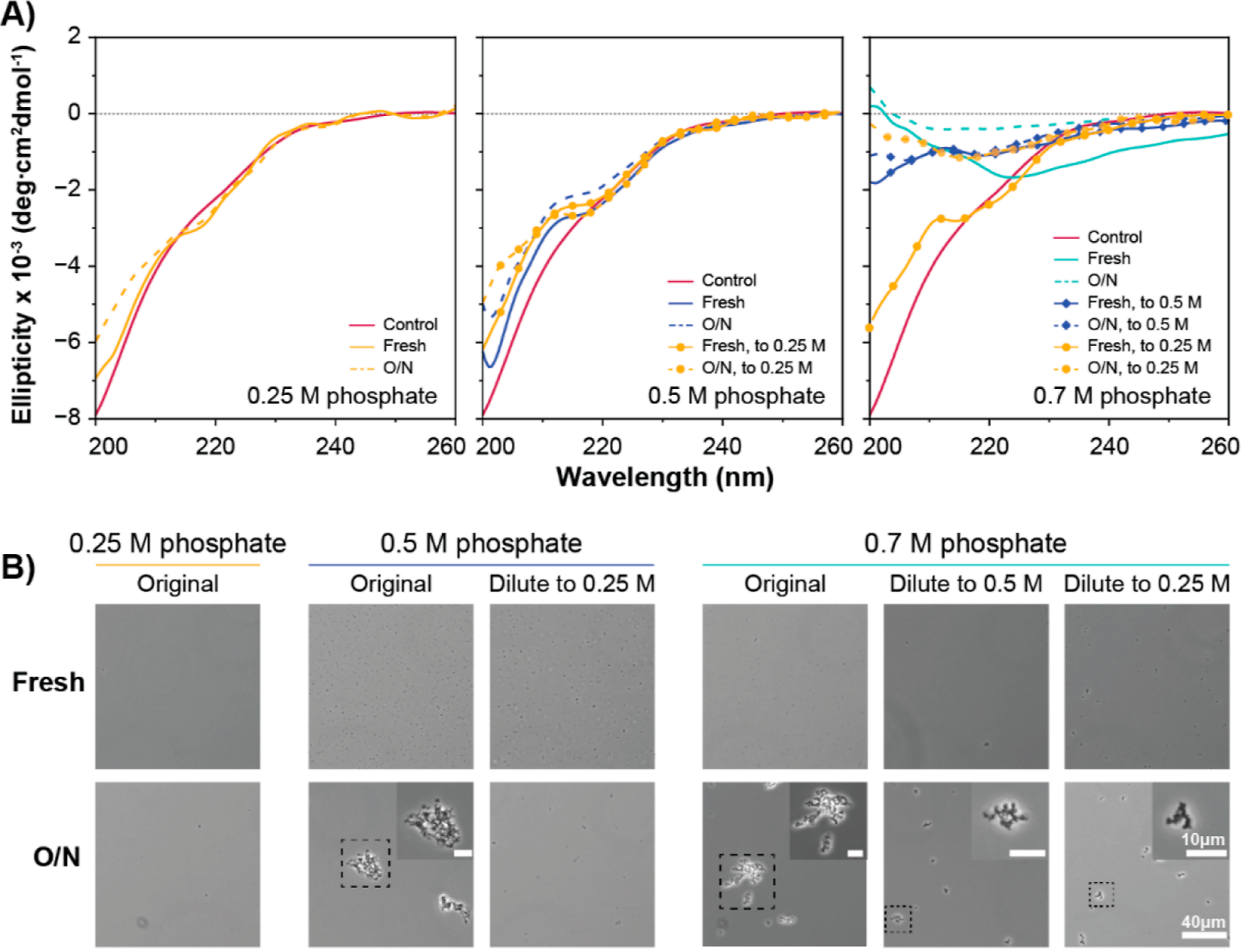
Reversibility of the phosphate-induced secondary structural change in SC-ADF3. (A) CD spectra of SC-ADF3 samples in 0.25, 0.5, and 0.7 M sodium phosphate (pH 7.4), including fresh (solid line) and overnight (O/N, dashed line) samples, as well as samples that were diluted from higher to 0.25 M (line with dots) and 0.5 M (line with diamond signs) phosphate. The control group was SC-ADF3 in 0 M sodium phosphate (red line). (B) Light microscopy images of the corresponding SC-ADF3 samples from the CD measurements. The scale bar is 40 μm for the normal images and 10 μm for the inset.

The reversibility of SC-ADF3 structural changes induced by phosphate was further studied using CD measurements and light microscopy (Figures 4, S6, and S7) at 22 °C. As also shown in previous results, SC-ADF3 had a combination of random coil and β-sheet structures in 0.5 M sodium phosphate and a primarily β-sheet structure in 0.7 M sodium phosphate. Diluting the SC-ADF3 samples, prepared in higher phosphate concentrations (0.5 or 0.7 M), to lower concentrations partially reverted the CD spectra to the level of the corresponding samples initially prepared at lower phosphate concentrations (Figure 4A). We also investigated whether the conformational change could reverse over time in 0.7 M sodium phosphate (Figure S7). Upon comparison with freshly prepared samples, the structural recovery capability diminished over time, and the conformational change became completely irreversible after 2 h of incubation in 0.7 M sodium phosphate. Furthermore, incubating SC-ADF3 in sodium phosphate overnight showed a decrease in the ellipticity signal while maintaining a similar spectral shape. The decrease in absolute molar ellipticity was likely attributed to protein aggregation that occurred over the extended time, which is also observable through light microscopy (Figure 4B).

### Higher Phosphate Concentration Led to a Stiffer Silk Protein Layer

**Figure 5:**
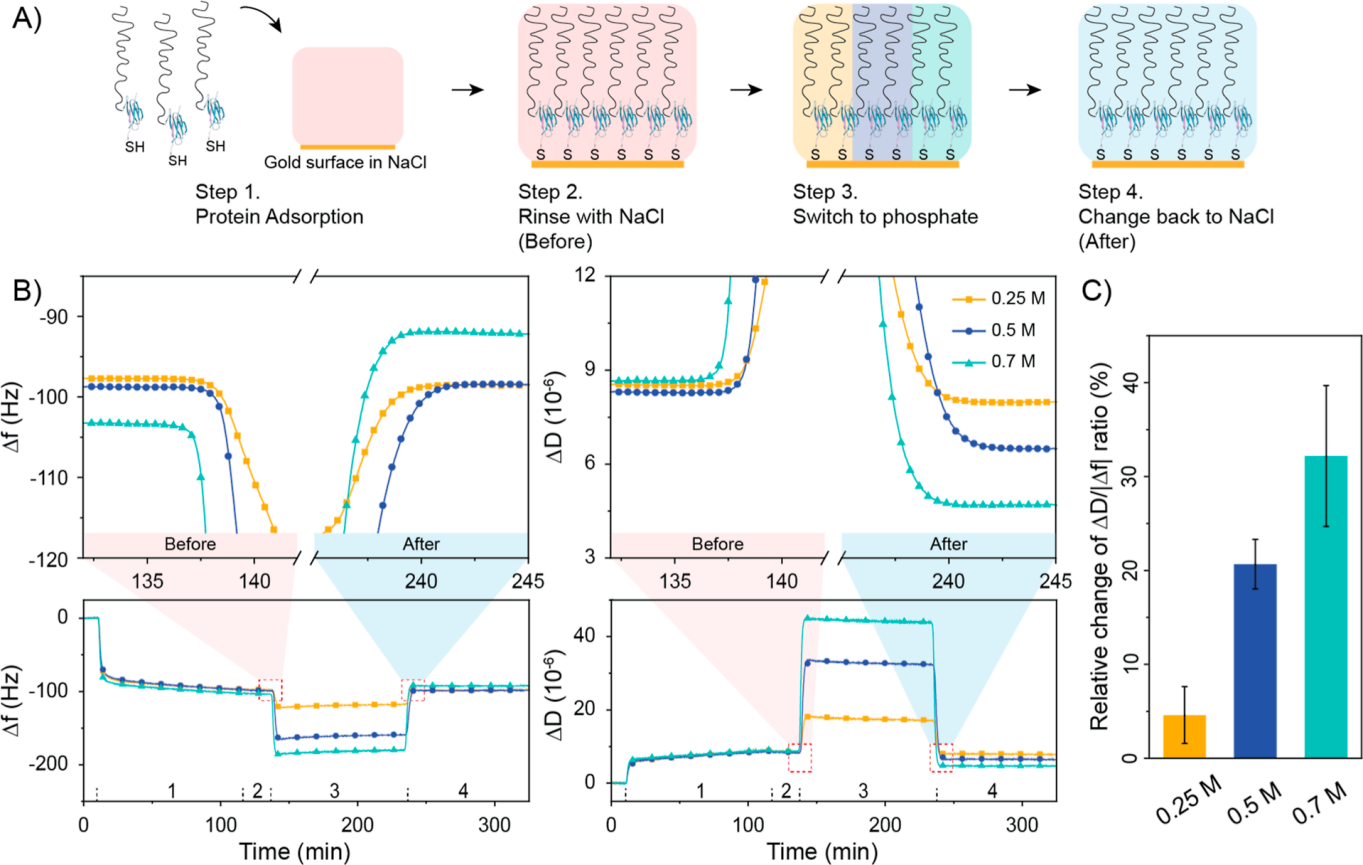
Changes in conformational and viscoelastic properties of SC-ADF3 at different phosphate concentrations. (A) Scheme of the QCM-D experimental steps. (B) Frequency and dissipation changes from QCM-D measurements (data shown for the third overtone). Upper row shows the zoomed region of frequency and dissipation changes before and after exposure to sodium phosphate. (C) Relative changes of the Δ*D*/|Δ*f*| ratio after exposure to 0.25, 0.5, and 0.7 M sodium phosphate, respectively. *N* = 3.

The conformational changes and viscoelastic properties of the SC-ADF3 proteins in 0.25, 0.5, and 0.7 M sodium phosphate were studied in real time using QCM-D (Figure 5). Prior to protein adsorption, a prestep of SpyCatcher–SpyTag ligation was conducted. This prestep allowed SC-ADF3 to maintain its natural and flexible state during protein adsorption, facilitated by the thiol group in ST-C, in contrast to the collapsing behavior observed in the absence of ST-C (Figure S8).

Protein adsorption was conducted in three separate chambers with 0.5 M sodium chloride to ensure uniform protein layers before the phosphate concentrations. The frequency changes (Δ*f*) in Figure 5B were around −100 Hz across chambers, indicating similar amounts of proteins and bound water adsorption. The adsorbed layers exhibited consistent dissipation changes (Δ*D*) of around 8.5 × 10^–6^ after protein adsorption, characterizing them as soft and viscoelastic layers. Subsequently, unbound proteins were washed using 0.5 M sodium chloride, and no further protein adsorption was observed, as indicated by unchanged frequency and dissipation. Switching to sodium phosphate led to a strong blank baseline shift in both frequency and dissipation due to varying ion concentrations (Figure S9), necessitating QCM-D data analysis in the same buffer as initial measurements (0.5 M sodium chloride) after exposure to phosphate.

Information on the conformational change and viscoelastic properties of the adsorbed layer can be obtained by calculating the value of the Δ*D*/|Δ*f*| ratio.^37−39^ To compare the effects of different concentrations of phosphate on the adsorbed protein layers, we calculated the relative changes in the Δ*D*/|Δ*f*| ratio after exposure to the phosphate solutions in Figure 5C. A small change (about 4.6%) for 0.25 M sodium phosphate suggested a minimal effect on the protein layer. In contrast, 0.7 M sodium phosphate had the largest impact, with the Δ*D*/|Δ*f*| ratio decreasing by approximately 32.2%, indicating a conformational change leading to increased stiffness. Besides, the Δ*f* value increased after exposure to 0.7 M sodium phosphate, suggesting the loss of bound water from the protein layer as it became stiffer. The change in the Δ*D*/|Δ*f*| ratio for 0.5 M sodium phosphate was moderate (20.7%) compared to the other concentrations, implying less conformational change compared to 0.7 M sodium phosphate. Additionally, the distribution of the dissipation curves from different overtones (Figure S10) could also provide insights into the properties of the adsorbed protein layers.^40^ The protein layer exposed to 0.7 M phosphate exhibited relatively low dissipation, and the responses across different overtones almost overlapped, suggesting that the adsorbed protein layer was rigid. These QCM-D results aligned with the CD measurements, indicating that SC-ADF3 exhibited minimal conformational and viscoelastic property changes at low phosphate concentration, while high phosphate concentration induced the β-sheet structure formation and a stiffer protein layer.

**Figure 6:**
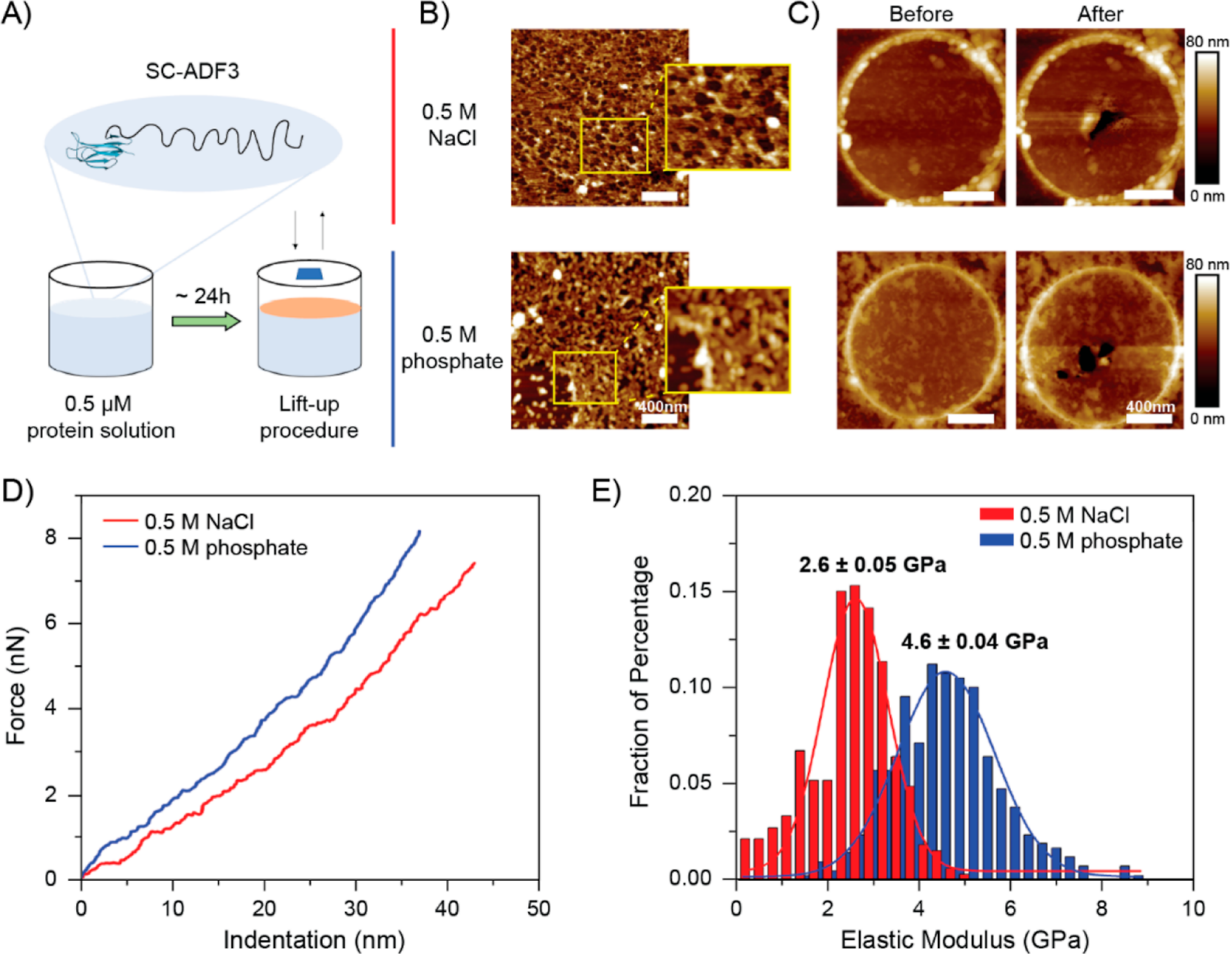
Comparison of the elastic modulus of SC-ADF3 in sodium chloride and sodium phosphate solutions. (A) Preparation method of supported and free-standing SC-ADF3 silk protein layers. (B) The corresponding AFM images of the protein layers were prepared in 0.5 M sodium chloride and 0.5 M sodium phosphate (pH 7.4) on the OTS-Si substrates. Scale bar: 400 nm. (C) AFM image of the protein layers on holey carbon TEM grids before and after the indentation experiments. Scale bar is 400 nm. (D) Exemplary F(δ) curves recorded for experiments carried out in 0.5 M sodium chloride (red, *N* = 326) and 0.5 M sodium phosphate (blue, *N* = 419). (E) Histograms of elastic moduli determined from fitting the *F*(δ) curves.

To further explore the correlation between the secondary structure and stiffness of the SC-ADF3 proteins in response to sodium phosphate, we determined their elastic moduli by performing AFM indentation experiments on SC-ADF3 layers. Due to protein aggregation observed at high sodium phosphate concentration in previous results, we selected 0.5 M sodium phosphate as the indentation experimental condition, with 0.5 M sodium chloride as a control. The protein layers were first self-assembled at the buffer–air interface and then transferred on the substrate of interest by a lift-up procedure (Figure 6A). In detail, transfer of the protein layers onto hydrophobic OTS-Si substrates was performed to gain insights into the morphology and thickness (Figures 6B and S2), while on the TEM grids, it was performed to determine the elastic modulus.

The AFM images on the OTS-Si substrates in the two different solutions revealed insights into the different packing of layers. In the presence of 0.5 M sodium chloride, the SC-ADF3 molecules exhibited more dense and interconnected structures, whereas in the presence of 0.5 M sodium phosphate, a minor tendency to form bundles and a more defined rod-like structure was observed.

For the indentation experiments, we recorded multiple indentation cycles on the free-standing part of the SC-ADF3 layers transferred onto holey TEM grids (Figure 6C). Indenting with a sufficient amplitude at the central point of the free-standing part of the film leads to a stretching of the film and thus allows for a determination of the elastic modulus of the film’s material via an appropriate model.^41,42^ From the AFM images recorded on various silk-covered holes, only those featuring a homogeneous layer were chosen to perform the AFM indentation experiments. Indentation curves (Figure 6D) were recorded and fitted using an elasticity model for films clamped onto circular holes to extract the elastic modulus according to eq 2. The Gaussian distributions of the two histograms presented in Figure 6E show a remarkable difference in the elastic modulus between 0.5 M sodium chloride and 0.5 M sodium phosphate. This difference could be conducible to the presence of β-sheet structures, as indicated by the CD measurements and QCM-D data, leading to increased stiffness of the layer.

### Presence of Phosphate at Higher Concentration Resulted in a Reduced Capability to Form New Interactions

**Figure 7:**
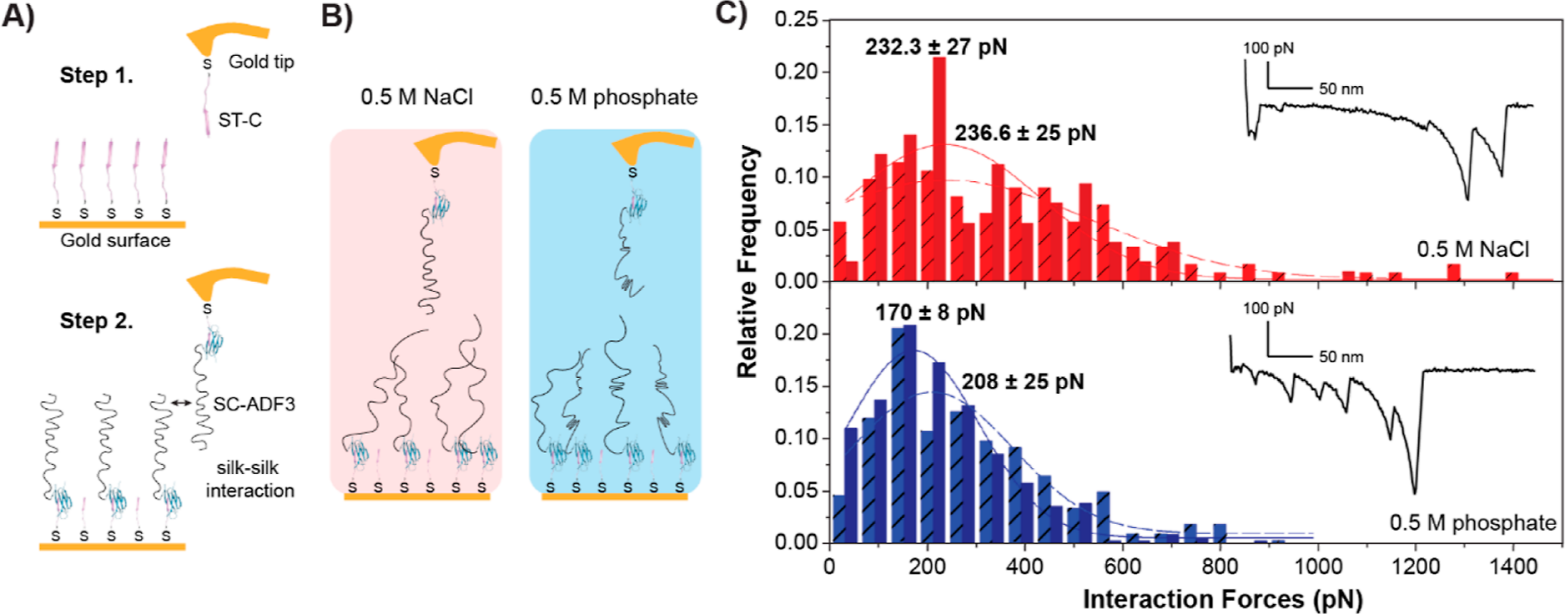
Interactions between SC-ADF3 proteins at the molecular level. (A) Schematic of tip and surface functionalization using a two-step process: first, ST-C peptides were bound to a gold surface, and then the SC-ADF3 proteins were covalently bound to the ST-C through the SpyCatcher–SpyTag interaction. (B) Illustration of the SMFS experiments carried out in 0.5 M sodium chloride and 0.5 M sodium phosphate (pH 7.4). (C) Interaction force histograms for 0.5 M sodium chloride (red *N* = 123 at 200 nm/s and light red with strips *N* = 107 at 500 nm/s) and 0.5 M sodium phosphate (blue *N* = 326 at 200 nm/s and light blue with strips *N* = 364 at 500 nm/s) with corresponding retraction force–distance curves.

The presence of β-sheets had a demonstrated impact on the viscoelasticity and on the stiffness properties of the protein layers, so intermolecular forces were probed by employing single-molecule force spectroscopy (SMFS) to study the interactions between SC-ADF3 proteins at the single molecular level. SC-ADF3 proteins were immobilized on both surface and on the AFM cantilever (Figure 7A), and multiple approaching and retraction cycles were recorded in the presence of 0.5 M sodium chloride and 0.5 M sodium phosphate (Figure 7B), respectively.

As shown in Figure 7C, the retraction curves, recorded at two different tip velocities, often exhibited multiple deadhesion peaks due to unbinding between multiple molecules attached to both surface and the tip. In particular, the recorded deadhesion forces, which correspond to the peak height of the retraction curves, reflect changes in the interaction forces depending on the salt conditions. From the histograms in Figure 7C, the force values recorded for the experiments carried out in the presence of 0.5 M sodium chloride were shifted to higher forces compared with those in the presence of 0.5 M sodium phosphate.

The slightly higher force for sodium chloride suggested stronger cohesion, possibly due to ADF3 silk chains being fully in random coil conformations. This is supported by the less frequent number of multiple deadhesion peaks observed in the presence of sodium chloride. In the presence of 0.5 M sodium phosphate, SC-ADF3 contained some β-sheet structures, which affected the interactions between the SC-ADF3 protein molecules, therefore resulting in a reduced capability to form strong bonds when placed in contact with other SC-ADF3 silk proteins.

## Discussion

Although phosphate ions recognizably have a significant influence on silk protein and fiber formation, the exact concentration of phosphate ions inside the spider gland remains unclear. According to the investigation of the major ampullate glands of *Nephila edulis* spider, O, P, S, Cl, Na, K, Ca, and Mg were the only elements detected from the specimens.^12^ The concentration of sodium chloride in the gland is estimated to be in the order of 0.1 to 0.15 M.^43^ Since the percentage composition of P was similar to that of Na, it can be inferred that the concentration of phosphate ions should be of the same order of magnitude as sodium chloride. Our *in vitro* results indicated that SC-ADF3 silk proteins were minimally affected by up to 0.35 M of phosphate, as determined by CD measurements. Additionally, QCM-D experiments showed that a concentration of 0.25 M phosphate resulted in only minor changes in the conformational and viscoelastic properties of the SC-ADF3 silk proteins. The difference in phosphate concentration observed between our study and the silk gland can be attributed to the fact that we solely focused on investigating the effects of phosphate, whereas there are other factors acting synergistically in the silk gland, such as pH, protein concentration, the presence of other proteins, differences in domain structure and protein size, and shear stress.^10,12,13,44^

In the absence of phosphate, ADF3-containing proteins predominantly exhibit random coil structures, a phenomenon also observed in other studies using CD spectroscopy.^20,45^ Chloride ions, unlike phosphate ions, possess less kosmotropic properties that enhance hydrophobic interactions and hydrogen bonding in molecules. As a result, in the absence of phosphate, SC-ADF3 silk proteins exhibit unstructured random coils due to the higher tendency of amino acid residues to interact with the surrounding solvent than with amino acids in the same or different backbones. The random coils can stretch via rotations around backbone bonds under external force, resulting in a reduced stiffness and elastic modulus of the SC-ADF3 silk protein layer, as observed by the QCM-D and AFM indentation experiments.

When phosphate is present, increased phosphate concentrations induce a transition in the secondary structure of SC-AFD3 silk proteins toward a predominant β-sheet conformation. Our CD results suggest that a lower concentration of sodium phosphate (below 0.35 M) had little effect on the secondary structure of SC-ADF3 silk proteins, and the proteins mainly exhibited random coils. The elevation in phosphate concentration (from 0.35 to 0.65 M) gradually induced the β-sheet formation; therefore, the SC-ADF3 silk proteins display both random coil and β-sheet structures in the CD spectra. Higher concentrations of phosphate (above 0.65 M) had a significant impact on the SC-ADF3 silk proteins, which predominantly presented a β-sheet conformation in the secondary structure. The QCM-D results further confirmed the conformational changes induced by the three distinct ranges of phosphate concentrations, as evidenced by an increase in the degree of conformational change with a corresponding rise in the phosphate concentration. Those findings align with several previous studies on various silk variants, including eADF3 and eADF4 repeats derived from *A. diadematus* dragline silk,^15,17,22^ major ampullate spidroin 1 from *Euprosthenops australis*,^46^ and major ampullate spidroin 2 (MaSp2) from *Nephila clavipes*,^14^ where increased phosphate concentrations induced the formation of β-sheet structures in silk proteins. Hence, although our study specifically examines the ADF3 protein, the influence of phosphate investigated herein is applicable to other silk protein variants.

Both our CD and QCM-D results indicate that the conformational changes caused by phosphate were irreversible over time, but at short incubation times, some reversion could occur. This may indicate a sequential cooperative mechanism. It was also observed in other studies that microspheres of eADF4 proteins induced by 1 M potassium phosphate with defined β-sheet structures were stable upon dilution,^15^ and spheres of MaSp2 proteins formed by the addition of 2 M potassium phosphate remained stable even after being dialyzed against water.^14^ Furthermore, we observed that prolonged incubation of SC-ADF3 silk proteins with a higher concentration of phosphate caused the aggregation of proteins, which was also observed in other studies.^10,16,45,46^ Due to the resolution limitation of light microscopy, we did not further investigate the properties of the aggregates. The irreversible conformational conversion from random coils to more β-sheets in SC-ADF3 silk proteins and the subsequent reduction in protein solubility were attributed to the kosmotropic properties of phosphate ions, which increased the intermolecular hydrophobic interactions^16,47^ and hydrogen bonding within the alanine-rich regions.^19^

The correlation between the conformational change, mechanical properties, and interactions of the SC-ADF3 silk proteins, such as stiffness, elastic modulus, and protein interactions, was explored using both QCM-D and AFM. The change of the Δ*D*/|Δ*f*| ratio from QCM-D experiments yielded the structural information on the adsorbed silk protein layer and showed that a higher concentration of phosphate led to a stiffer protein layer. The AFM indentation experiment further verified that the presence of 0.5 M phosphate resulted in a higher elastic modulus of the SC-ADF3, indicating greater stiffness in the presence of phosphate. A study on repeats derived from the major ampullate spidroin 1 from *N. clavipes* by using solution-state NMR spectroscopy also revealed similar findings, where an increase in phosphate concentration resulted in a decrease in the local flexibility of the repeats.^18^ Furthermore, the impact of the conformational changes on the interactions among SC-ADF3 silk proteins was investigated using SMFS, which shows a reduced capability of forming new interactions between proteins in the presence of phosphate. Therefore, the phosphate-induced conformational conversion from random coils to β-sheet structures in SC-ADF3 silk proteins increased stiffness and reduced protein interactions, resulting in decreased deformability and a reduced capability of forming new bonds between adjacent molecules of the SC-ADF3 silk proteins.

## Conclusions

Our results show the impacts of different concentrations of phosphate on the conformational change in the SC-ADF3 silk proteins, with these changes subsequently affecting the stiffness and interactions of the proteins. These changes were likely due to the kosmotropic properties of phosphate ions, which increased the level of intermolecular hydrophobic interactions and triggered the formation of β-sheets. Our results contribute to understanding why the SLCs of eADF3-containing silk proteins induced by phosphate could not form fibers. The formation of β-sheets in the eADF3-containing silk proteins affected the interactions between the proteins; therefore, the SLCs induced by phosphate were less deformable and irreversible.^25^ Conversely, the interactions of random coil structural molecules in the absence of phosphate had higher cohesion forces and interconnects, thereby enabling the production of functional materials, such as fibers.^25^

Furthermore, we have confirmed that the SpyCatcher–SpyTag system, incorporated with a thiol group, can successfully be used to study SC-ADF3 silk proteins at the molecular level. Although this study primarily focuses on the effect of phosphate, the same system can be applied to investigate other factors and more complex conditions.^48^ We expect that this work will offer valuable insights into improving the preparation of silk proteins for high-performance artificial silk materials.

## Supporting Information Available

Protein and peptide amino-acid sequences; SDS-PAGE analysis of SC-ADF3 protein; AFM images and corresponding height profiles of the protein layers; FTIR absorbance spectra of SC-ADF3; CD analysis of SC-ADF3 and SC; comparison of CD spectra of CBM-ADF3-CBM and SC-ADF3; scheme of the reversibility study of secondary structure changes with different incubation times; results of the reversibility study with different incubation times; QCM-D measurements of the SC-ADF3 protein without ST-C; QCM-D measurements of the blank solutions; and comparison of the 3, 5, 7, 9, and 11 overtones from the QCM-D measurements (PDF)
