# Supplementary material for: Effect of Phosphate on the Molecular Properties, Interactions, and Assembly of Engineered Spider Silk Proteins
Source: Biomacromolecules. 2024 Jun 25;25(7):3990–4000. doi: 10.1021/acs.biomac.4c00115 (PMC11238326; doi:10.1021/acs.biomac.4c00115)
Supplement: Supplementary file 1 — bm4c00115_si_001.pdf [file bm4c00115_si_001.pdf]

Supporting Information

The effect of phosphate on the molecular properties, interactions, and assembly of engineered spider silk proteins

Yin Yin, Alessandra Griffo, Karin Jacobs, Hendrik Hähl, Adrián Gutiérrez Cruz, Markus B. Linder*

Protein and peptide amino-acid sequences

SC-ADF3:

MGAMVDTLSGLSSEQGQSGDMTIEEDSATHIKFSKRDEDGKELAGATMKLRDSSGKTISTWISDGQVK

DFYLYPGKYTFVETAAPDGYEVATAITFTVNEQGQVTVNGKATKGDAHISASASASAGASAAASAGAGA

GAGPGQQGPGQQGPGQQGPYGPGASAAAAAAGGYGPGSGQQGPSQQGPGQQGPGGQGPYGPG

ASAAAAAAGGYGPGSGQQGPGGQGPYGPGSSAAAAAAGGNGPGSGQQGAGQQGPGQQGPGGSA

AAAAGGYGPGSGQQGPGQQGPGGQGPYGPGASAAAAAAGGYGPGSGQGPGQQGPGGQGPYGP

GASAAAAAAGGYGPGSGQQGPGQQGPGQQGPGGQGPYGPGASAAAAAAGGYGPGYGQQGPGQQ

GPGGQGPYGPGASAASAASGGYGPGSGQQGPGQQGPGGQGPYGPGASAAAAAAGGYGPGSGQQG

PGQQGPGQQGPGQQGPGGQGPYGPGASAAAAAAGGYGPGSGQQGPGQQGPGQQGPGQQGPGQ

QGPGQQGPGQQGPGQQGPGQQGPGGQGAYGPGASAAAGAAGGYGPGSGQQGPGQQGPGQQG

PGQQGPGQQGPGQQGPGQQGPGQQGPYGPGASAAAAAAGGYGPGSGQQGPGQQGPGQQGPGG

QASASASAAASAASTVANSLEHHHHHH

ST-C (purchased from Biomatik):

AHIVMVDAYKPTKGGC

Supporting figures

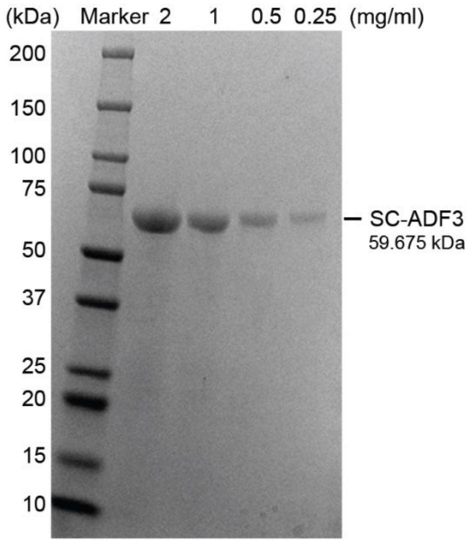

Figure S1. Sodium dodecyl sulfate–polyacrylamide gel electrophoresis (SDS-PAGE) analysis of SC-ADF3 protein in different concentrations showing the purity of the sample.

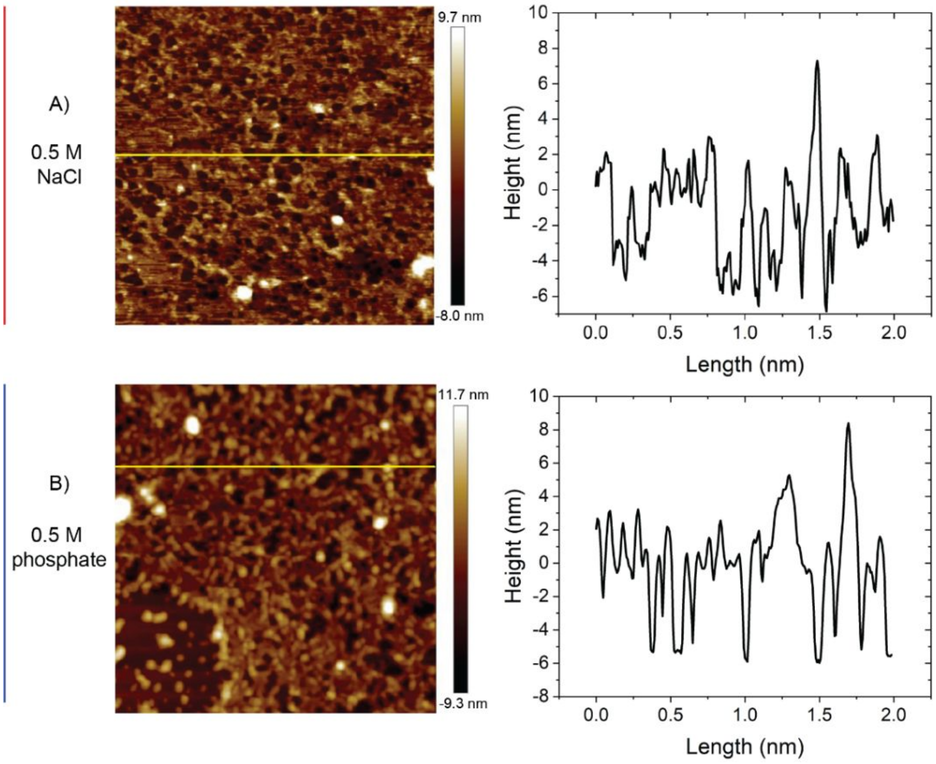

Figure S2. AFM images and corresponding height profiles of the protein layers. The protein layers were prepared in (A) 0.5 M NaCl and (B) 0.5 M sodium phosphate (pH 7.4) on OTS-Si substrates. The position of the height profile indicated by the yellow horizontal line.

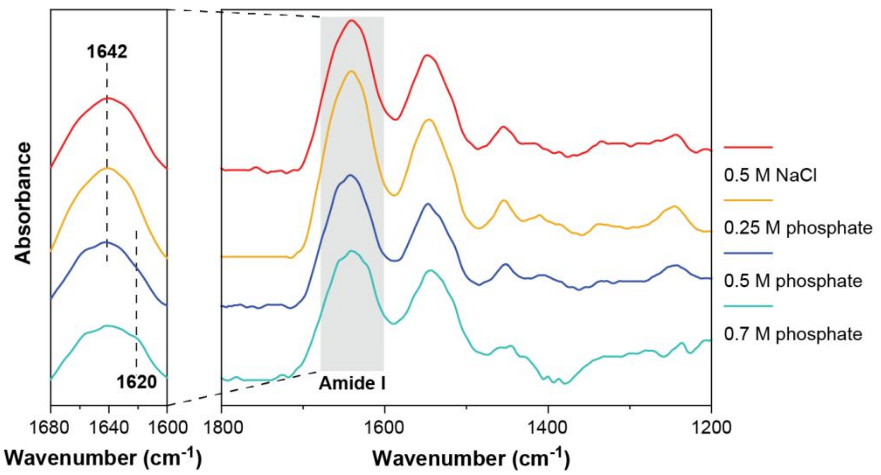

Figure S3. Fourier-transform infrared spectroscopy (FTIR) absorbance spectra were recorded for 8 mg/ml SC-ADF3 in 0.5 M sodium chloride and 0.25 M, 0.5 M, and 0.7 M sodium phosphate after incubation at room temperature for 1.5 h. All the samples were scanned with 32 accumulations in absorbance mode within the wavenumber range of 1800-1200 cm^{-1}, at a resolution of 4 cm^{-1}. A shoulder at 1620 cm-1 was observed in the 0.7 M phosphate sample, indicating the presence of β-sheet structure. These findings are consistent with previous studies of β-sheet structures on silk proteins[1–3].

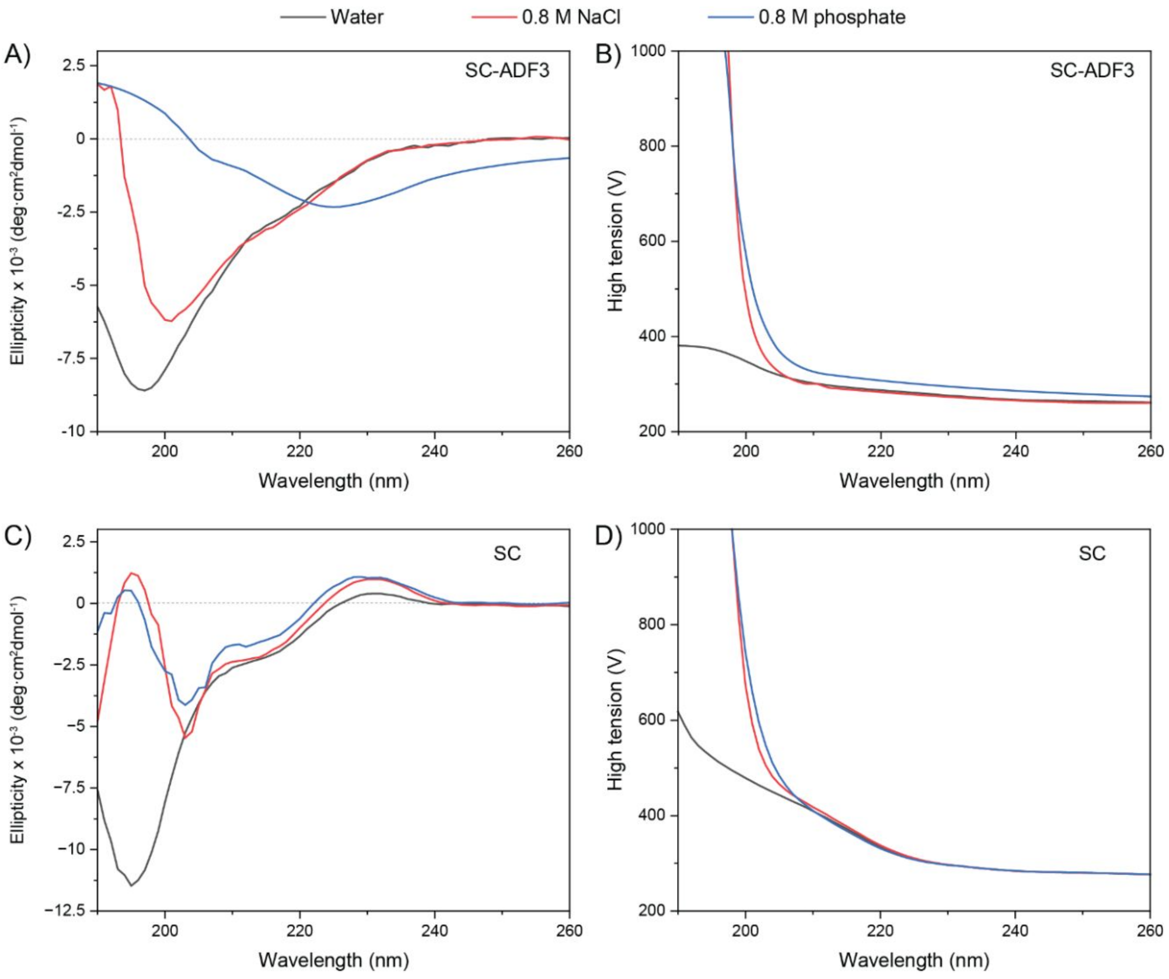

Figure S4. CD analysis of SC-ADF3 and SC. (A, B) CD spectra and high-tension voltage measured during CD analysis of 0.1 mg/ml SC-ADF3 in water, 0.8 M sodium chloride and 0.8 M sodium phosphate, pH 7.4. (C, D) CD spectra and high-tension voltage measured during CD analysis of 0.1 mg/ml SC in water, 0.8 M sodium chloride and 0.8 M sodium phosphate, pH 7.4. Due to the saturated high tension (HT>700 V) for samples in 0.8 M sodium chloride and 0.8 M sodium phosphate, the signal below 200 nm could not be clearly observed. Normally, such high concentrations of chloride ions and phosphate ions absorb strongly below 200 nm, therefore, wavelengths between 200 and 260 nm were selected as the range for other CD analysis.

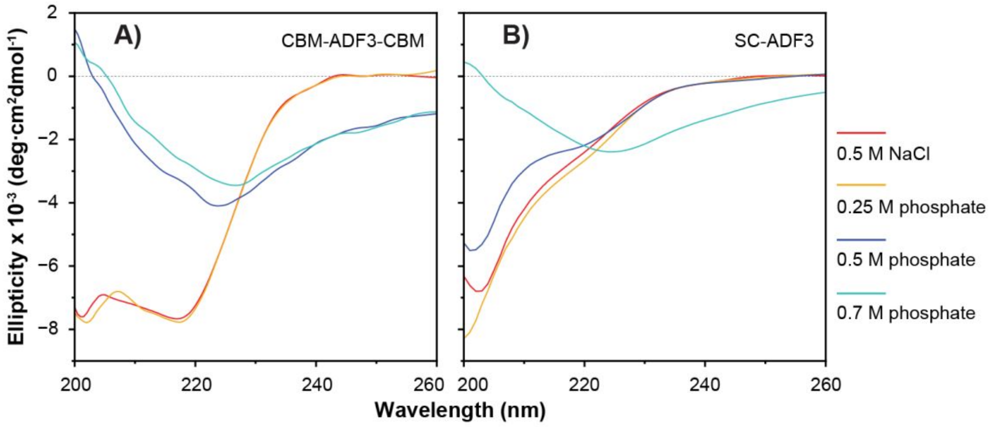

Figure S5. The comparison of CD spectra of (A) CBM-ADF3-CBM and (B) SC-ADF3 in different concentration of sodium phosphate (pH 7.4) at 22°C.

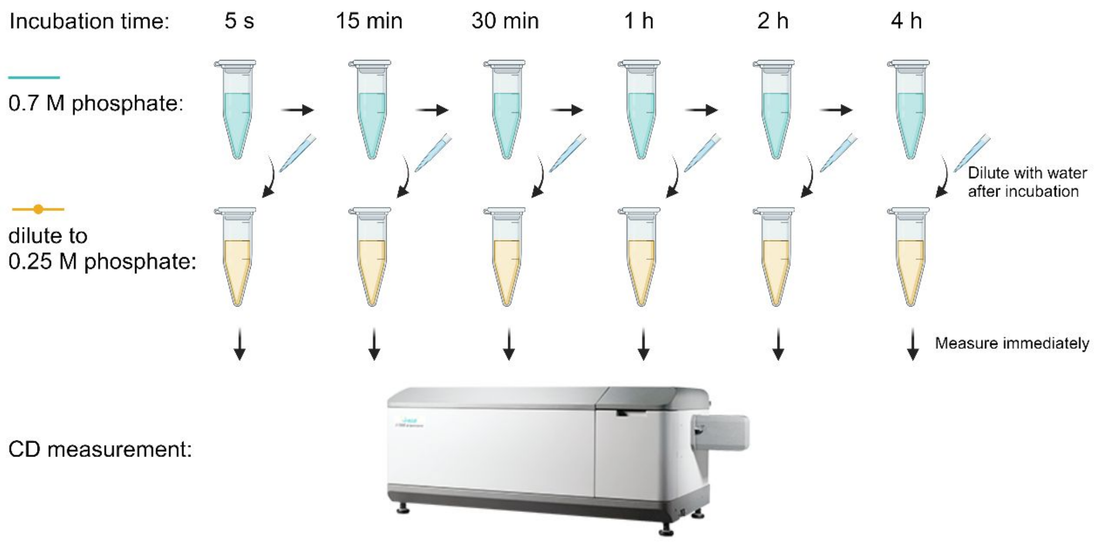

Figure S6. Scheme of the CD experimental steps for the reversibility study of secondary structure change with different incubation time. The 0.7 M phosphate group included SC-ADF3 (0.28 mg/ml) in 0.7 M sodium phosphate. Dilute to 0.25 M phosphate group refers to SC-ADF3 samples diluted from the 0.7 M phosphate group to a final concentration of 0.1 mg/ml SC-ADF3 and 0.25 M sodium phosphate. Samples from the control and 0.7 M phosphate group were incubated for a specific duration and then measured. Samples from the dilute to 0.25 M phosphate group were diluted from the 0.7 M phosphate group after a specific incubation period in phosphate, followed by immediate measurement after dilution. -Control group consisted of SC-ADF3 in water without phosphate.

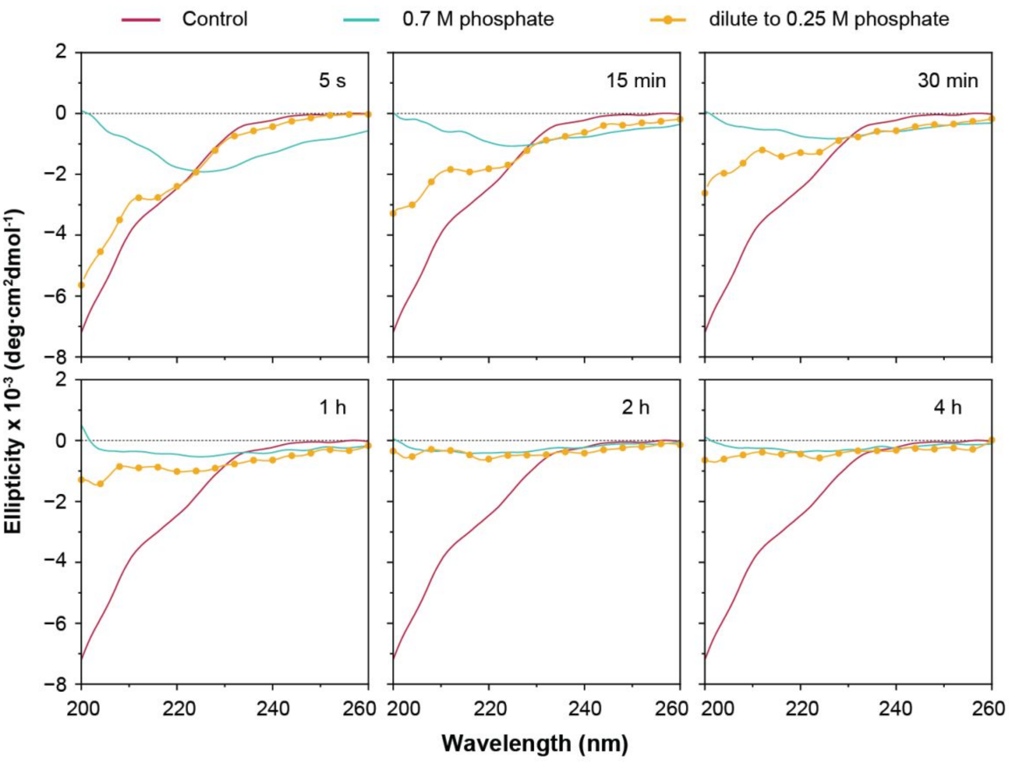

Figure S7. CD spectra of SC-ADF3 samples incubated in 0.7 M sodium phosphate (pH 7.4) for freshly prepared 5 s, 15 min, 30 min, 1 h, 2 h and 4h (green line), as well as corresponding samples that were diluted to 0.25 M sodium phosphate (yellow line with dots). The control group was SC-ADF3 sample in 0 M sodium phosphate (red line).

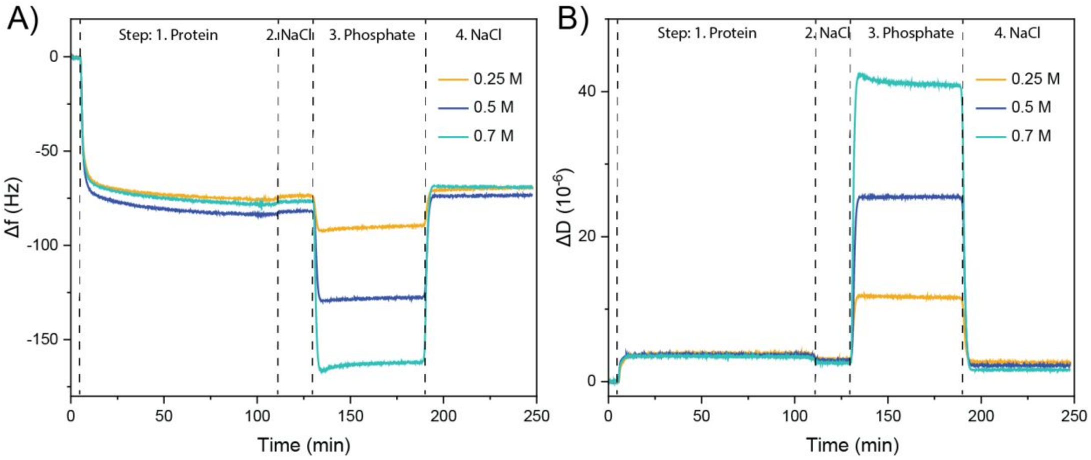

Figure S8. QCM-D measurements of SC-ADF3 protein without ST-C. (A, B) Frequency and dissipation change in the QCM-D measurements for SC-ADF3 proteins with 0.25 M, 0.5 M, and 0.7 M sodium phosphate, pH 7.4. The observed dissipation changes in the layers formed after protein absorption in all chambers were approximately 3 x 10^{-6}, suggesting that the protein layers were rigid and likely underwent collapse on the surface.

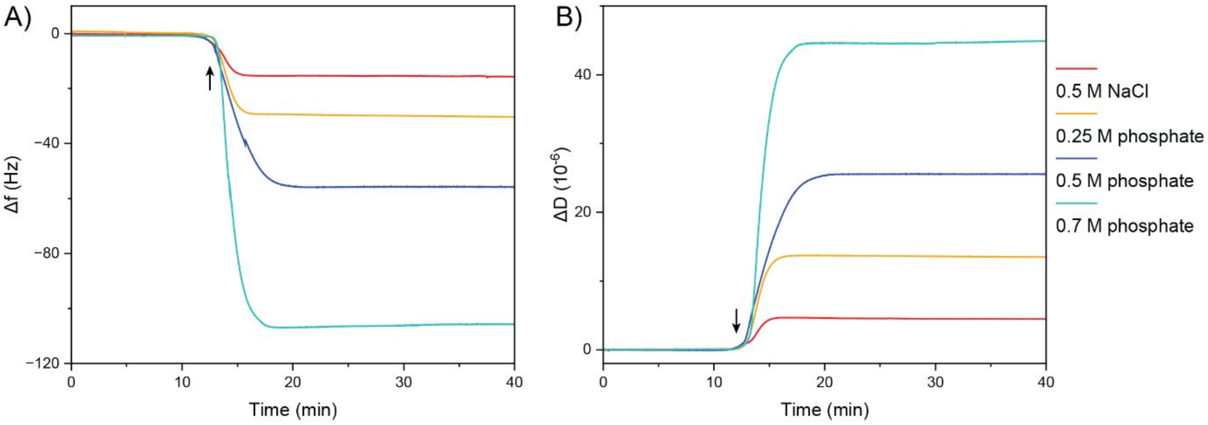

Figure S9. QCM-D measurements of the blank solutions. (A, B) Frequency and dissipation change in the QCM-D measurements of the blank solutions: 0.5 M sodium chloride, 0.25 M, 0.5 M, and 0.7 M sodium phosphate, pH 7.4. The arrows indicate the point of loading for each of the solutions.

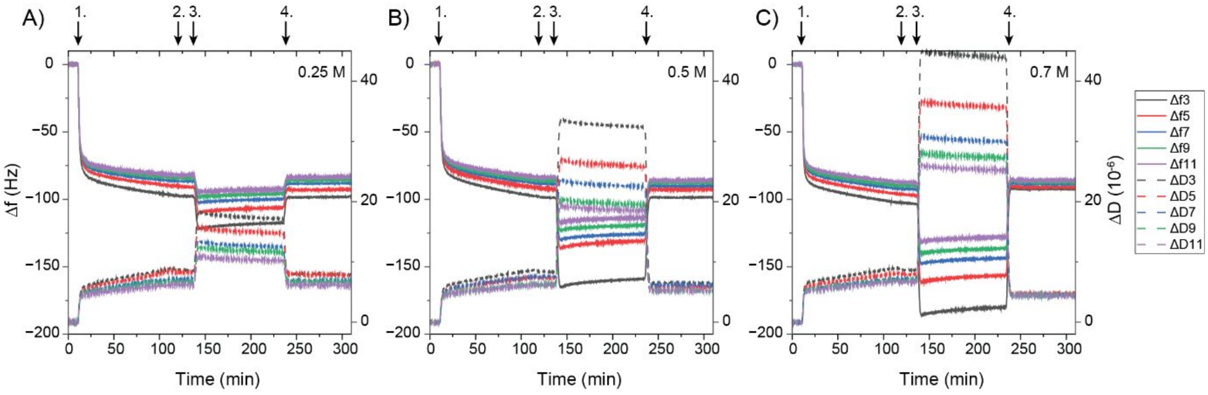

Figure S10. Comparing the 3, 5, 7, 9 and 11 overtones from the QCM-D measurements. The frequency and dissipation change from the same QCM-D measurements shown in Figure 5 in the presence of (A) 0.25 M, (B) 0.5 M and (C) 0.7 M sodium phosphate. The numbers and arrows indicate the procedure: 1. protein adsorption, 2. rinse with 0.5 M sodium chloride, 3. switch to different concentrations of phosphate and 4. change back to 0.5 M sodium chloride.

Reference

(1) Mohammadi, P.; Jonkergouw, C.; Beaune, G.; Engelhardt, P.; Kamada, A.; Timonen, J. V. I.; Knowles, T. P. J.; Penttila, M.; Linder, M. B. Controllable Coacervation of Recombinantly Produced Spider Silk Protein Using Kosmotropic Salts. *Journal of Colloid and Interface Science* **2020**, *560*, 149–160. https://doi.org/10.1016/j.jcis.2019.10.058.

(2) Arndt, T.; Jaudzems, K.; Shilkova, O.; Francis, J.; Johansson, M.; Laity, P. R.; Sahin, C.; Chatterjee, U.; Kronqvist, N.; Barajas-Ledesma, E.; Kumar, R.; Chen, G.; Strömberg, R.; Abelein, A.; Langton, M.; Landreh, M.; Barth, A.; Holland, C.; Johansson, J.; Rising, A. Spidroin N-Terminal Domain Forms Amyloid-like Fibril Based Hydrogels and Provides a Protein Immobilization Platform. *Nat Commun* **2022**, *13* (1), 4695. https://doi.org/10.1038/s41467-022-32093-7.

(3) Andersson, M.; Jia, Q.; Abella, A.; Lee, X.-Y.; Landreh, M.; Purhonen, P.; Hebert, H.; Tenje, M.; Robinson, C. V.; Meng, Q.; Plaza, G. R.; Johansson, J.; Rising, A. Biomimetic Spinning of Artificial Spider Silk from a Chimeric Minispidroin. *Nat Chem Biol* **2017**, *13* (3), 262–264. https://doi.org/10.1038/nchembio.2269.
